# The role of rodents in the ecology of *Ixodes ricinus* and associated pathogens in Central and Eastern Europe

**DOI:** 10.3389/fcimb.2013.00056

**Published:** 2013-10-01

**Authors:** Andrei D. Mihalca, Attila D. Sándor

**Affiliations:** Department of Parasitology and Parasitic Diseases, University of Agricultural Sciences and Veterinary Medicine, Cluj-Napoca, Romania

**Keywords:** rodents, *Ixodes ricinus*, Europe, ecology, synchronous activity

## Abstract

Rodents comprise more species than any other mammal order. Most rodents are considered keystone species in their ecological communities, hence the survival of many other species in the ecosystem depend on them. From medical point of view, this is particularly important for rodent-dependent pathogens. In the particular case of tick-borne diseases, rodents are important as hosts for vector ticks and as reservoir hosts (Lyme borreliosis, human granulocytic anaplasmosis, Crimean-Congo hemorrhagic fever, Tick-borne relapsing fevers, tick-borne rickettsioses, babesiosis). Community and population ecology of rodents was shown to be correlated with disease ecology in the case of many tick-borne diseases. In Eastern Europe, several adult hard-tick species use rodents as their principal hosts: *Ixodes apronophorus*, *I. crenulatus*, *I. laguri*, *I. redikorzevi, I. trianguliceps*. However, the majority of ticks feeding on rodents are immature stages of ticks which as adults are parasitic on larger mammals. Larvae and nymphs of *Ixodes ricinus*, the most abundant and medically important tick from Europe, are commonly found on rodents. This is particularly important, as many rodents are synanthropic and, together with other micromammals and birds are often the only available natural hosts for ticks in urban environments. This work reviews the correlated ecology of rodents and *I. ricinus*.

## Background

Vector-borne diseases generally have a very complex ecology, and various factors are influencing their distribution, natural prevalence, seasonal, and multiannual dynamics. As compared to other hematophagous arthropods, hard-ticks have a particular biology, as they take only one, very large meal per life stage. As most hard-ticks follow a three-host life cycle this means that during a complete generation, each individual tick will feed on three individual hosts. Hence, the contact between a potential vector tick and a susceptible/reservoir host is limited to maximum three per generation. As a consequence, each acquired pathogen is transmitted to a subsequent host by the following developmental stage of the tick. Therefore, the prerequisite for vectorial competence in ixodid ticks is the transstadial maintenance and/or transovarial transmission of infection (Randolph, 2004).

From medical and social points of view, tick-borne pathogens are particularly important if they are able to infect humans. However, not all known tick-borne pathogens are recognized as human pathogens. This does not necessarily mean that they are not theoretically infectious to humans, but certain ecological factors are rather limiting the possibility of transmission to humans. Although in some areas of the world the number of tick species recorded on humans is relatively high (Estrada-Peña and Jongejan, 1999; Dantas-Torres et al., 2012), usually the vast majority of them belong to very few species. In South America, the most important human ticks are certain species of the genus *Amblyomma* (Dantas-Torres et al., 2012), in Turkey it is the genus *Hyalomma* (Bursali et al., 2010), while in Central and Eastern Europe the majority of ticks collected from humans are *Ixodes ricinus* (Briciu et al., 2011). In Central and Eastern Europe, *I. ricinus* is the dominant tick on humans, birds and rodents in forest environment (Briciu et al., 2011; Mihalca et al., 2012a, b, c). *I. ricinus* is the most generalist tick of Europe, with more than 300 host species reported (Anderson, 1991).

However, the importance of these hosts in the ecology of tick-borne diseases is variable and depends on certain factors: (i) the abundance of hosts during the activity peaks of ticks; (ii) the availability of the host for questing ticks; (iii) the possibility of an individual host to harbor simultaneously multiple developmental stages (i.e., larvae and nymphs); (iv) the capacity of the hosts to maintain and transmit the pathogens to ticks; (v) the capacity of the host to allow bridging of pathogens between ticks, both intraspecifically and interspecifically.

The density of questing *I. ricinus* is influenced by various factors, among which the local abundance of hosts is among the most important (Randolph et al., 2002). Additionally, the prevalence of infection with certain pathogens in ticks depends on the ecological pattern of tick infestation in reservoir hosts. Because of their abundance, proven role as reservoir hosts for important human pathogens and host for ticks, rodents represent a good epidemiological model for the study of disease ecology. Hence, our review will focus on the role of rodents in the ecology of *I. ricinus*. Population ecology and distribution of certain rodent species seems to be connected to the epidemiological situation in humans, mainly in the case of certain tick-borne pathogens as *Borrelia burgdorferi* sensu lato or tick-borne encephalitis virus (TBE).

## Large and uncommon or small and abundant?

There are various direct and experimental evidences which show that larger hosts have higher level of infestation with macroparasites. These size-dependent differences in parasitism intensity can be found both between hosts from different species as well as between individuals form the same species (Randolph, 2004). The factors incriminated are physical, behavioral or immunological and act individually or synergistically (Randolph, 2004). Larger hosts (e.g., deer, fox), which are usually carrying many ticks, are generally found in much lower densities than rodents. On the other hand, even if intensity of tick parasitism on small rodents is much smaller, their abundance certainly compensates (Randolph, 2004). Hence, overall small and abundant rodents might account for hosting the majority of local tick population in any given time. Rodents in temperate areas show a cyclic population fluctuation, which has usually yearly cycles (in contrast to the multiyear boreal rodent cycles), with most species in Central European region having the population maxima in late summer/early autumn (Krebs, 2013). This peak in abundance overlaps with the density peak observed in many areas for subadult stages (especially nymphs) in Europe, of questing *I. ricinus* (Randolph et al., 2002). Moreover, this is a high mobility period of most rodent species, thus the hosts transport the resistant, but abundant developmental stage of the tick life-cycle. Population minima in most rodents occurs in early spring, a period overlapping with the highest abundance of adult stages, which do not require rodent host, but generally parasitize larger mammals (species lacking such yearly population fluctuations). In this way there is a synchronization in *I. ricinus* development and rodent population densities in temperate European forest rodents, making these later optimal hosts for this tick species.

Various rodents were reported as hosts for *I. ricinus* in Central and Eastern Europe. A comprehensive list of hosts include: *A. agrarius, A. flavicollis, A. sylvaticus, A. uralensis, Arvicola amphibius, Chionomys nivalis, Cricetus cricetus, Dryomys nitedula, Eliomys quercinus, Glis glis, Micromys minutus, Microtus agrestis, M. arvalis, M. subterraneus, M. tatricus, Mus musculus, Muscardinus avellanarius, Myodes glareolus, Rattus norvegicus, Sciurus vulgaris* (Nosek and Sixl, 1972; Matuschka et al., 1991; Mihalca et al., 2012b; Pérez et al., 2012).

However, several studies showed that certain rodent species are more commonly used as hosts by *I. ricinus*. In Germany, among the most important hosts for larvae and nymphs are *A. flavicollis*, *A. agrarius*, and *M. glareolus* (Matuschka et al., 1991). In France (L'Hostis et al., 1996) and Romania (Mihalca et al., 2012b) the main host for immature stages was *M. arvalis*. *A. uralensis* and *A. sylvaticus* were also reported to be important hosts for larvae and nymphs of *I. ricinus* in Romania (Mihalca et al., 2012b). On the other hand, although certain species of rodents are generally or locally abundant (*M. musculus*, *M. spicilegus*, *R. norvegicus*), they rarely harbor *I. ricinus*, and if they do so, the intensity is usually low (Paulauskas et al., 2009; Mihalca et al., 2012b). This pattern may be related to the differences in home ranges and activity patterns of these species, with *Mus* species tending to hold small home ranges in comparison to most *Apodemus* species (Krebs, 2013), while most *Microtus* voles have their daily activity peaks overlapping with the most active periods of questing ticks (Randolph et al., 2002).

While this correlation of the peak tick activity with the synchronous abundance of rodent populations is common, there are species-specific differences observed, which may be caused by differences in host ecology. Matuschka et al., (1991) showed that certain hosts (i.e., *A. agrarius*) tend to be more heavily parasitized by nymphal *I. ricinus* than others (i.e., *M. glareolus* or *A. flavicollis*) because of the synchronous host-tick abundance. Additionally, they also suggested that during the summer, when specific rodent abundance is locally low and the questing ticks are still active, they prefer to feed on lizards which might impair the transmission cycle of *B. burgdorferi* s.l. Pérez et al., (2012) noted a discrepancy between the seasonal activity patterns of questing ticks (measured by flagging) and the tick abundance on sympatric rodents.

Another important factor in the ecology of tick-borne pathogens is the correlation of peak activities of larvae and nymphs. Rodents are particularly important as some species are able to host both stages, synchronously. This is crucial for the natural maintenance of pathogens like TBEV, which produce non-systemic infections and it is maintained in the rodent host only for short time. Field studies have shown this in rodents which are able to host together larvae and nymphs of *I. ricinus* (Matuschka et al., 1991; Randolph et al., 1999; Mihalca et al., 2012b). This is uncommon in other host categories, like mesocarnivores (Széll et al., 2006) or large herbivores (Ruiz-Fons and Gilbert, 2010).

## What happens after the tick detaches from a rodent?

After feeding, each developmental stage of the tick detaches. Nevertheless, the duration of feeding and survival rates of detached ticks is dependent on various factors, and the host species is among the most important. Post larval bite acquired resistance to *I. ricinus* has been described in *M. glareolus* (Dizij and Kurtenbach, 1995). This results in reduced engorged weight and reduced survival in nymphs. The same study showed that post\textit-bite immunity for *I. ricinus* was not present in *A. flavicollis*. Similarly, *A. sylvaticus* was shown to develop significantly lower acquired immunity to *I. trianguliceps* than laboratory mice (Randolph, 1979, 1994). Immunity of rodents to ticks is also dependent on the level of sexual hormones. In *M. glareolus* and *A. sylvaticus*, the individuals with high testosterone levels showed reduced innate and acquired resistance to feeding *I. ricinus* (Hughes and Randolph, 2001). However, a recent study found no difference between the tick burdens of different sexes of two commonly parasite rodent species, *A. sylvaticus* and *M. glareolus* (Kiffner et al., 2011). Moreover, several experimental studies showed that certain rodent species are more infective to ticks than others. Pérez et al. found that in the case of *B. burgdorferi* s.l., *M. glareolus* was more infective to ticks than *A. sylvaticus* (Pérez et al., 2012). When infectivity to *I. ricinus* was assessed for *B. afzelii*, *M. glareolus* was still more infective than *A. sylvaticus* and *A. flaviocollis* (Humair et al., 1999). When two species of genus *Apodemus* (*A. flavicollis* and *A. sylvaticus*) were compared for their infectivity with *B. burgdorferi* s.l. to *I. ricinus*, no significant differences were found (Gern et al., 1994).

## Concluding remark

Rodents are one of the most important hosts for the maintenance of a number of *Ixodes* species in Central and Eastern Europe and crucial in shaping the population dynamics of *I. ricinus*. The spatial and temporal synchrony of temperate forest rodents and the parasitizing *I. ricinus* developmental stages, create optimal conditions not only for high abundance of the ticks, but for pathogen transmission for important diseases. This makes rodent species the key players in the yearly cycle of a number of important diseases, like Lyme borreliosis or TBE.

### Conflict of interest statement

The authors declare that the research was conducted in the absence of any commercial or financial relationships that could be construed as a potential conflict of interest.
